# Supplementary material for: Gamma entrainment induced by deep brain stimulation as a biomarker for motor improvement with neuromodulation
Source: Nat Commun. 2025 Mar 26;16:2956. doi: 10.1038/s41467-025-58132-7 (PMC11947250; doi:10.1038/s41467-025-58132-7)
Supplement: Supplementary file 1 — Supplementary Information [file 41467_2025_58132_MOESM1_ESM.pdf]

# SUPPLEMENTARY MATERIAL

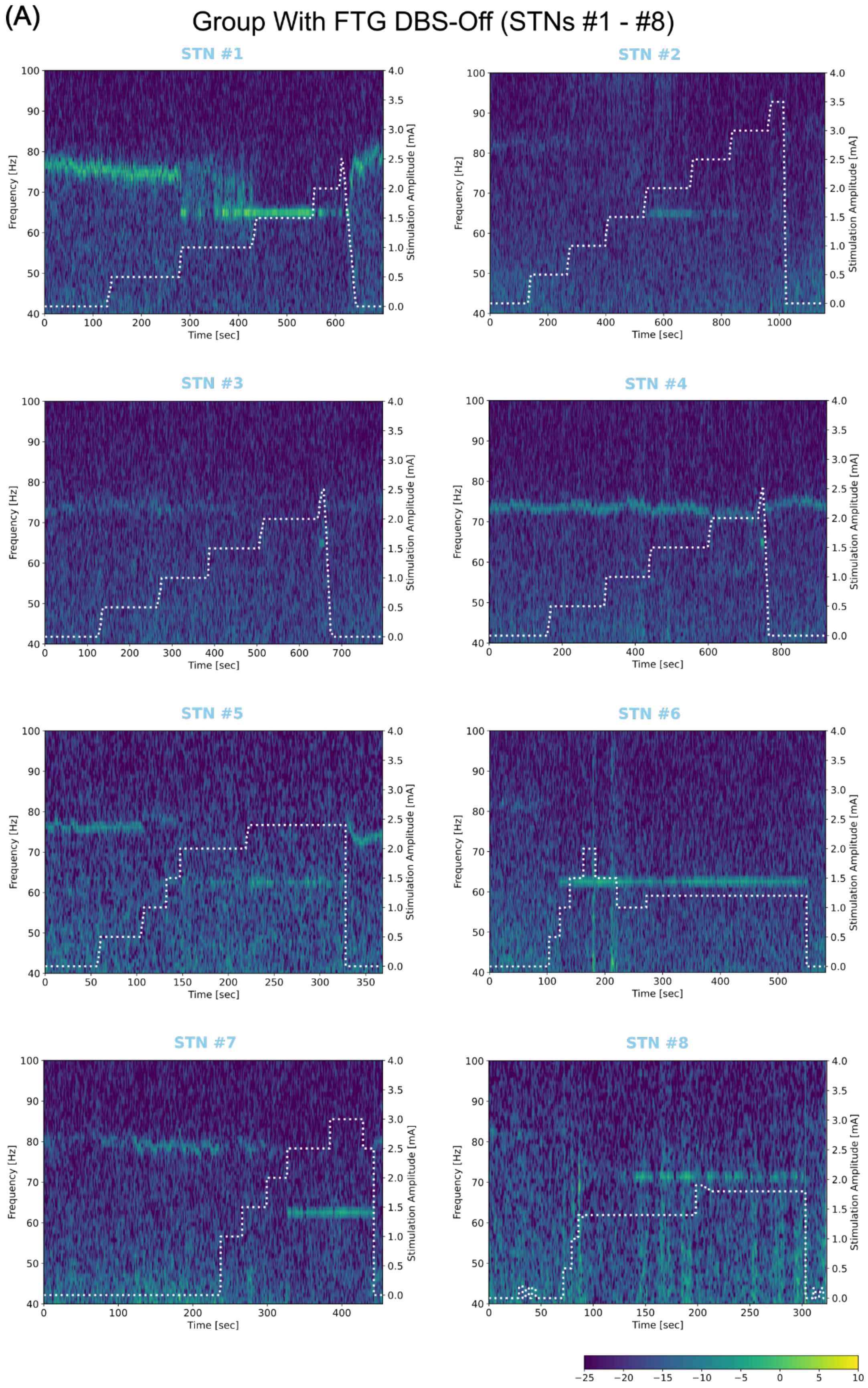

**(B)** Group Without FTG DBS-Off (STNs #9 - #15)

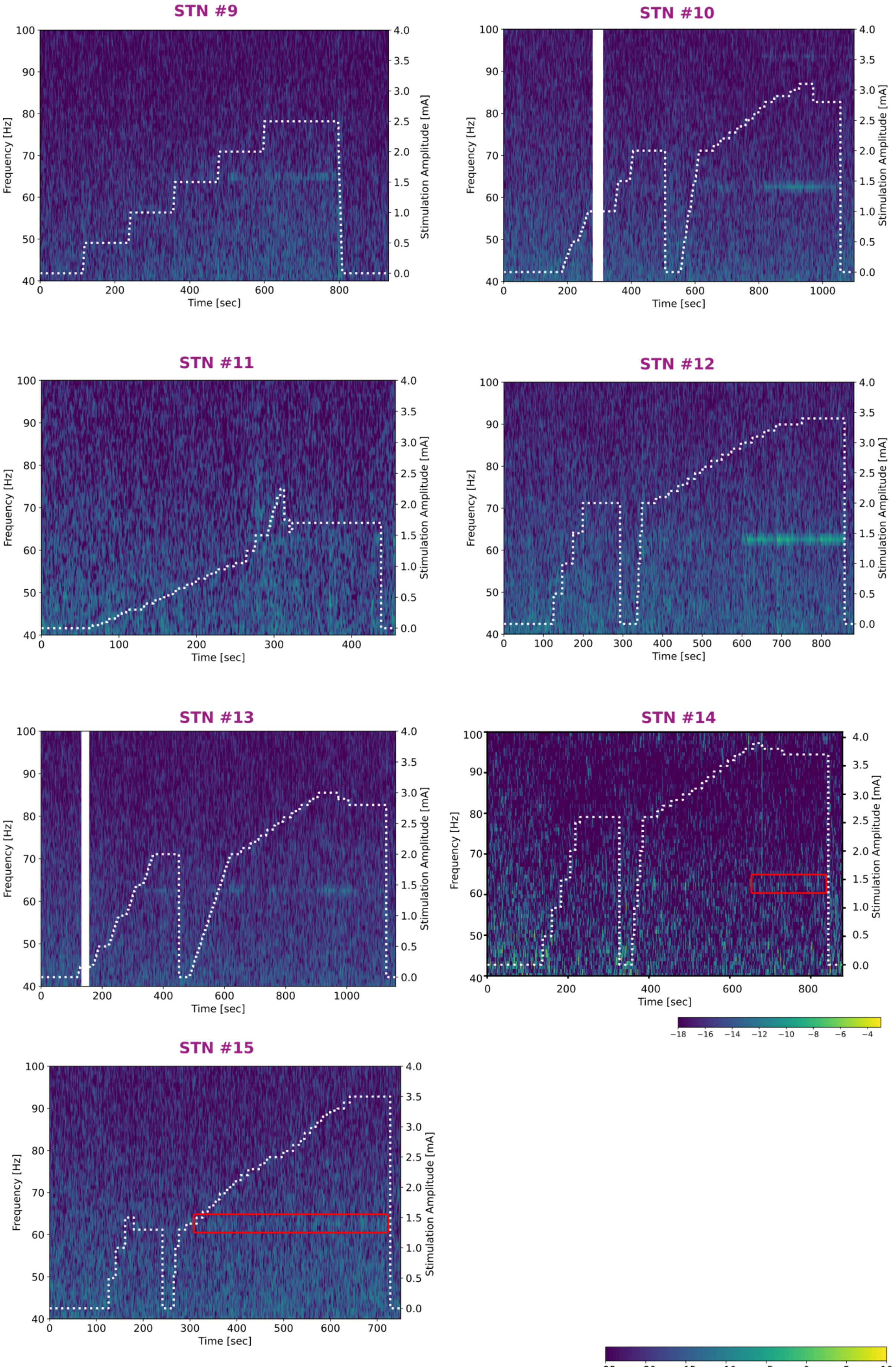

(C) Group with no Gamma Activity (STNs #16 - #19)

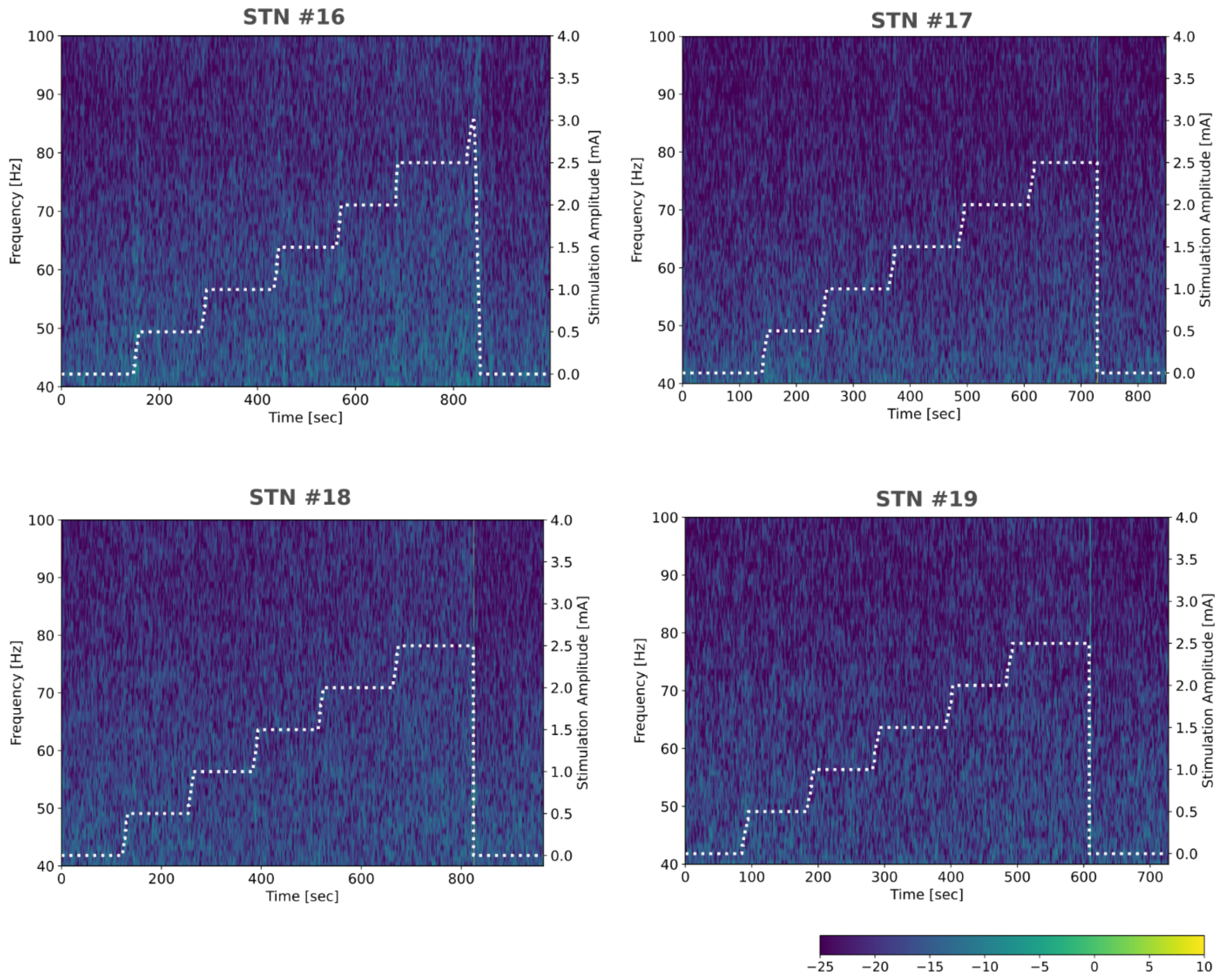


**Supplementary Figure 1. Individual spectrograms of all STNs.** (A) STNs with spontaneous FTG during DBS-off. (B) STNs without spontaneous FTG during DBS-off. Note that figure of the STN #14 has a different color scale. (C) STNs without gamma activity detected on/off DBS.
